# Crimean-Congo Hemorrhagic Fever Virus Seroprevalence in Human and Livestock Populations, Northern Tanzania

**DOI:** 10.3201/eid3004.231204

**Published:** 2024-04

**Authors:** Ellen C. Hughes, William de Glanville, Tito Kibona, Blandina Theophil Mmbaga, Melinda K. Rostal, Emanuel S. Swai, Sarah Cleaveland, Felix Lankester, Brian J. Willett, Kathryn J. Allan

**Affiliations:** University of Liverpool Institute of Infection Veterinary and Ecological Sciences, Liverpool, UK (E.C. Hughes);; University of Glasgow College of Medical Veterinary and Life Sciences, Glasgow, Scotland, UK (E.C. Hughes, W. de Glanville, S. Cleaveland, K.J. Allan);; Washington State University, Pullman, Washington, USA (E.C. Hughes, F. Lankester);; Nelson Mandela African Institute of Science and Technology, Arusha, Tanzania (T. Kibona);; Global Animal Health Tanzania, Arusha, Tanzania (T. Kibona, F. Lankester);; Kilimanjaro Christian Medical University College, Kilimanjaro, Tanzania (B.T. Mmbaga);; EcoHealth Alliance, New York, New York, USA (M.K. Rostal);; Ministry of Agriculture Livestock and Fisheries, Dodoma, Tanzania (E.S. Swai);; MRC-University of Glasgow Centre for Virus Research, Glasgow, UK (B.J. Willet)

**Keywords:** Crimean-Congo hemorrhagic fever virus, viruses, vector-borne infections, tickborne diseases, zoonoses, hemorrhagic fever, public health surveillance, livestock, Tanzania

## Abstract

We conducted a cross-sectional study of Crimean-Congo hemorrhagic fever virus (CCHFV) in northern Tanzania. CCHFV seroprevalence in humans and ruminant livestock was high, as were spatial heterogeneity levels. CCHFV could represent an unrecognized human health risk in this region and should be included as a differential diagnosis for febrile illness.

Crimean-Congo hemorrhagic fever virus (CCHFV) is a tickborne orthonairovirus with potential to cause severe Crimean-Congo hemorrhagic fever (CCHF) disease in humans, which can lead to human-to-human transmission (*1*). CCHFV is a World Health Organization priority pathogen for research and development (*2*). Although a wide range of wild and domestic animals can be infected (*3*), CCHFV does not typically cause clinical disease in nonhuman species (*1*). In eastern Africa, intermittent outbreaks of CCHF disease in humans have occurred in Uganda since 2013 (*4*), but the epidemiology of CCHFV remains poorly understood. Northern Tanzania, neighboring Uganda, has been identified as an area likely to be at high risk for human disease caused by CCHFV, because competent tick vectors and suitable environmental conditions exist in the region (*5*), but no clinical CCHF cases have yet been reported in the country.

To investigate CCHFV exposure in northern Tanzania, we performed serologic testing on human and ruminant livestock serum samples collected in 2016 during an investigation of several zoonotic pathogens (*6*) (Appendix). The study used a multilevel sampling frame of 351 humans and 7,456 randomly selected livestock in linked households in Arusha and Manyara Regions (Figure). We tested serum samples by using the ID Screen CCHF Double Antigen Multi-species ELISA (IDvet, https://www.innovative-diagnostics.com) (Appendix). We estimated seroprevalence by using the Survey package in R (The R Foundation for Statistical Computing, https://www.r-project.org) (*7*). We assessed species-level differences in seroprevalence by using a mixed-effects model with household and village as random effects. We investigated patterns of spatial autocorrelation in village-level seroprevalence by using the Moran *I* statistic and assessed correlation of village-level seroprevalence between species pairs by using the Pearson correlation coefficient (ρ) (Appendix).

**Figure:**
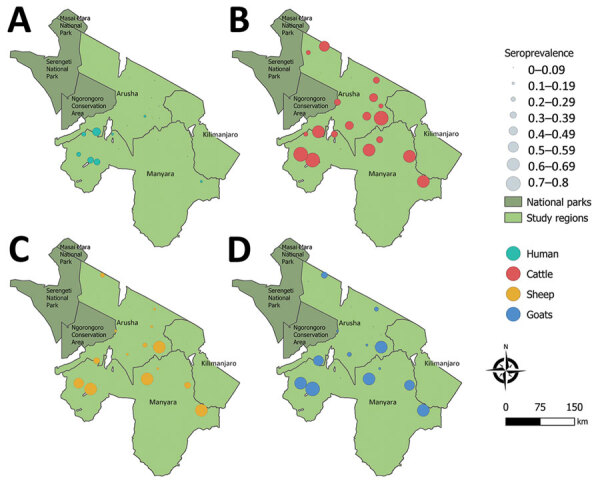
Sampling area for study of seroprevalence of Crimean-Congo hemorrhagic fever virus in human and livestock populations, northern Tanzania. Circles indicates seroprevalence rates for humans (A), cattle (B), sheep (C), and goats (D). The pictured region is near Uganda, where human Crimean-Congo hemorrhagic fever cases have been documented (*4*).

Overall, seroprevalence was high in all livestock species: cattle 49.6% (95% CI 40.0%–59.2%), goats 33.8% (95% CI 21.7%–47.5%), sheep 27.8% (95% CI 17.0%–40.6%) (Table; Figure). Sheep and goats had significantly lower odds of exposure than cattle: sheep OR was 0.32 (95% CI 0.27–0.37, p<0.001) and goats OR 0.45 (95% CI 0.39–0.51; p<0.001). Village-level seroprevalence ranged widely in all species but values were consistent with those reported elsewhere in East Africa (*3*) (Table). The finding of higher seroprevalence in cattle than in sheep and goats is also consistent with other settings in Africa (*3*) and might reflect differences in host feeding preferences of *Hyalomma* spp. ticks, considered chief vectors of CCHFV (*1*). However, further work is required to understand the relative contribution of different host species to viral maintenance, and their relationship to human infection risk.

**Table:** Seroprevalence of Crimean-Congo hemorrhagic fever virus in human and livestock populations, northern Tanzania*

Species	No. tested	Overall seroprevalence (95% CI)	Seroprevalence range per village (95% CI)		Moran *I* statistic (p value)
Low	High
Cattle	3,015	49.6 (40.0–59.2)	5.3 (1.2–9.4)	76.6 (70.3–82.8)	−0.09 (0.60)
Sheep	2,059	27.8 (17.0–40.6)	0.0 (0–3.9)	70.3 (55.5–85.0)	−0.09 (0.57)
Goats	2,382	33.8 (21.7–47.5)	0.0 (0–5.8)	79.6 (68.3–90.8)	−0.10 (0.61)
Human	351	15.1 (8.5–23.8)	0.0 (0.0–16.1)	50 (30.7–69.2)	0.43 (0.001)

*Serum samples were collected in northern in 2016 and tested for antibodies to Crimean-Congo hemorrhagic fever virus. Moran I statistic and associated p value are shown for the village level (Appendix).

Overall, human seroprevalence was 15.1% (95% CI 11.7%–19.2%), but village-level seroprevalence varied widely between study sites (Table). Seroprevalence was similar to that reported in health-care-seeking patients in Kenya in 2012 (*8*), but higher than the 1.2% seroprevalence reported in community participants elsewhere in Tanzania (*9*). However, interpretation of those regional comparisons is challenging in light of the substantial observed between-village variation in our study (Table).

Assessment of spatial autocorrelation via Moran *I* statistic showed no evidence of village-level spatial autocorrelation in livestock (Table), suggesting that although context-specific drivers, such as husbandry practices and local agroecology are likely involved, drivers of exposure were not observable at this scale. In contrast, we observed significant positive spatial autocorrelation in the village-level human seroprevalence (Moran *I* statistic 0.43; p<0.001) and clustering of higher seroprevalence villages in the western part of Manyara (Figure). In addition, species-pair correlations showed that village-level human and livestock seroprevalence were not well correlated (cattle, ρ = 0.34, p = 0.142; sheep, ρ = 0.35, p = 0.13; goats, ρ = 0.42, p = 0.062), and we saw high human seroprevalence in some low livestock seroprevalence locations and vice versa (Appendix). That heterogeneity, combined with differences in spatial distribution, could suggest different drivers of exposure in livestock and human populations. However, discrepancies in sample size could exaggerate those differences, so further linked investigation into human and livestock exposure and patterns of tick infection are required. Further exploration of specific risk factors is ongoing and could provide clarity on drivers of exposure.

The high human exposure levels to CCHFV implies that clinical CCHF is a potentially serious, underdiagnosed health risk in this population and suggests that CCHF should be included as a differential diagnosis for undifferentiated febrile illness in northern Tanzania. However, evidence of human seropositivity in the absence of clinical cases is common, even where health professionals are familiar with CCHF diagnosis (*8*,*10*). The causes of disease emergence in such populations are poorly understood, and further research into regions like northern Tanzania, where the virus is endemic but human disease has not been reported, is critical to understanding human disease risk.

In conclusion, we found that CCHFV is circulating widely in livestock across northern Tanzania. CCHFV seroprevalence in the region shows high spatial heterogeneity and further investigations are needed to understand drivers of exposure. In addition, high human seroprevalence demonstrates widespread exposure of the population to CCHFV and suggests that CCHF should be included as a differential diagnosis for febrile illness in this region.

Appendix Additional information on seroprevalence of Crimean-Congo hemorrhagic fever virus in human and livestock populations, northern Tanzania.
