# Supplementary material for: Crimean-Congo Hemorrhagic Fever Virus Seroprevalence in Human and Livestock Populations, Northern Tanzania
Source: Emerg Infect Dis. 2024 Apr;30(4):836–8. doi: 10.3201/eid3004.231204 (PMC10977851; doi:10.3201/eid3004.231204)
Supplement: Appendix — Additional information on seroprevalence of Crimean-Congo hemorrhagic fever virus in human and livestock populations, northern Tanzania. [file 23-1204-Techapp-s1.pdf]

Article DOI: https://doi.org/10.3201/eid3004.231204

EID cannot ensure accessibility for supplementary materials supplied by authors. Readers who have difficulty accessing supplementary content should contact the authors for assistance.

Crimean-Congo Hemorrhagic Fever Virus Seroprevalence in Human and Livestock Populations, Northern Tanzania

Appendix

Study Design

Samples were collected as part of a cross-sectional sero-survey undertaken in 2016 to investigate several zoonotic pathogens, including *Brucella* spp., *Coxiella burnetii* and Rift Valley Fever Virus (RVFV), in livestock and humans across a range of agricultural systems (*1*,*2*). This study was designed to achieve a range of aims with a target of 400 households to estimate a household-level prevalence of 50% for multiple outcomes, with around 5% error at the 95% confidence interval. Livestock sample sizes at the household-level were based on a desire to detect at least one seropositive animal of each species (cattle, goat, sheep) in the household with 95% herd sensitivity if the herd was "exposed," given an expected within-herd prevalence of target diseases of 40%, and assumed diagnostic test sensitivity of 90% and specificity of 100%. This meant a target of 10 randomly selected animals of each species per household, or sampling all animals present where herd/flock sizes of a particular species were 10 or less (*3*). Animals less than 6 months were excluded. Livestock samples were collected using a multilevel sampling approach and resulted in a total of 3,015 cattle, 2,382 goats, and 2,059 sheep sampled from 417 households across 20 villages. Villages were randomly selected using generalized random tessellation stratified sampling to ensure spatial balance (*2*,*4*). Human serum samples (n = 351) were collected from a random selection of 113 of these households across 17 villages, as well as four additional village sites without linked livestock samples. In these households, all willing people aged five or above were sampled. GPS coordinates were not recorded for sites where livestock sampling did not also occur, so these do not appear on the maps (Figure).

Laboratory Testing

Samples from all species were heat-treated at 56°C for two hours in Tanzania before shipping to the UK (license no. TARP(S)2016/49) for analysis at the MRC-University of Glasgow Centre for Virus Research using a commercially produced, species-independent, double antigen sandwich ELISA (IDvet, Grabels, France). Manufacturer-reported sensitivity and specificity values for the ID Screen® CCHF Double Antigen Multi-species (IDvet, Grabels, France) are shown in Appendix Table 1. Further details of the ELISA can be found in Sas et al. 2018 (*5*) and discussion of potential cross-reactivity in Hughes 2022 (*6*).

Cross-reactivity with related orthonairoviruses is a potential issue with serologic testing for CCHFV, although recent large-scale studies have suggested limited evidence of cross-reactivity (*7*,*8*). In livestock, the performance of the ID Screen® CCHF Double Antigen Multi-species (IDvet, Grabels, France) has been shown to correlate highly with results obtained using other methods for CCHF antibody detection, including immunofluorescent assays, in-house indirect species-specific ELISAs against both glycoprotein Gc and nucleoprotein (NP) (*9*,*10*). The assay has also been used in the testing of human samples (*11*), including in conjunction with other commonly used human-specific assays to retrospectively confirm the earliest case of CCHF in Spain, published in EID in 2021 (*12*).

Statistical Analysis

Seroprevalence was calculated for each species both at a population and village-level. Overall seroprevalence and 95% confidence intervals were calculated using the *Survey* package in R, using village and household as cluster identifiers (village = primary sampling unit, household = secondary sampling unit) (*13*). Village-level seroprevalence was calculated as the number of positives/total number sampled in each village with binomial confidence intervals.

Village-level seroprevalence for all species was plotted on maps of the study area. Maps were created in QGIS version 3.16.0 (*14*). All statistical analyses were performed in R statistical environment, version 3.6.1 (*15*). Mixed-effects logistic regression models were implemented using the *lme4* package (*16*). Random effects were included at the village and household level. Moran's I was calculated using the *spdep* package in R (*17*). A Moran's I statistic of 1 is equivalent to perfect spatial clustering, while a value of −1 represents perfect dispersal.

Statistical significance was set at $p \leq 0.05$. Moran's I statistic was calculated using the village level seroprevalence and did not include analysis of co-variables. As such, it is a test for spatial autocorrelation of seroprevalence for the village context only, and does not account for specific variables that may drive exposure within village units.

Ethics

The study protocols, questionnaires, and consent documents were approved by the Kilimanjaro Christian Medical Centre (KCMC) (approval no. 832) and National Institute of Medical Research (NIMR) (approval no. 2028) ethics committees, and University of Glasgow Medical, Veterinary and Life Sciences (MVLS) Ethics Committee (approval no. 200140152). Permission to carry out the study in Tanzania was provided by the Tanzania Commission for Science and Technology (permit no. 2014–244-ER-2005–141). Written informed consent or assent for sample collection and questionnaire administration was collected from all participants. Samples were imported into the UK under license TARP(S)2016/49. Permission to publish was granted by the Director of Veterinary Services, Tanzania (Act No. 17 of 2003).

References

1. Herzog CM, de Glanville WA, Willett BJ, Kibona TJ, Cattadori IM, Kapur V, et al. Pastoral production is associated with increased peste des petits ruminants seroprevalence in northern Tanzania across sheep, goats and cattle. Epidemiol Infect. 2019;147:e242. PubMed https://doi.org/10.1017/S0950268819001262

2. de Glanville WA, Davis A, Allan KJ, Buza J, Claxton JR, Crump JA, et al. Classification and characterisation of livestock production systems in northern Tanzania. PLoS One. 2020;15:e0229478–0229478. PubMed https://doi.org/10.1371/journal.pone.0229478

3. Cameron AR, Baldock FC. A new probability formula for surveys to substantiate freedom from disease. Prev Vet Med. 1998;34:1–17. PubMed https://doi.org/10.1016/S0167-5877(97)00081-0

4. Semango G, Hamilton CM, Kreppel K, Katzer F, Kibona T, Lankester F, et al. The sero-epidemiology of *Neospora caninum* in cattle in northern Tanzania. Front Vet Sci. 2019;6:327. PubMed https://doi.org/10.3389/fvets.2019.00327

5. Sas MA, Comtet L, Donnet F, Mertens M, Vatansever Z, Tordo N, et al. A novel double-antigen sandwich ELISA for the species-independent detection of Crimean-Congo hemorrhagic fever

virus-specific antibodies. Antiviral Res. 2018;151:24–6. PubMed https://doi.org/10.1016/j.antiviral.2018.01.006

6. Hughes EC. Serological investigations of emerging viral zoonoses: Crimean-Congo haemorrhagic fever virus in Tanzania and severe acute respiratory syndrome coronavirus 2 in Scotland. Dissertation. Glasgow: University of Glasgow; 2022.

7. Grech-Angelini S, Lancelot R, Ferraris O, Peyrefitte CN, Vachiery N, Pédarrieu A, et al. Crimean-Congo hemorrhagic fever virus antibodies among livestock on Corsica, France, 2014–2016. Emerg Infect Dis. 2020;26:1041–4. PubMed https://doi.org/10.3201/10.3201/eid2605.191465

8. Hartlaub J, Daodu OB, Sadeghi B, Keller M, Olopade J, Oluwayelu D, et al. Cross-reaction or co-infection? Serological discrimination of antibodies directed against Dugbe and Crimean-Congo hemorrhagic fever orthonairovirus in Nigerian Cattle. Viruses. 2021;13:1398. PubMed https://doi.org/10.3390/v13071398

9. Balinandi S, von Brömssen C, Tumusiime A, Kyondo J, Kwon H, Monteil VM, et al. Serological and molecular study of Crimean-Congo hemorrhagic fever virus in cattle from selected districts in Uganda. J Virol Methods. 2021;290:114075. PubMed https://doi.org/10.1016/j.jviromet.2021.114075

10. Belij-Rammerstorfer S, Limon G, Maze EA, Hannant K, Hughes E, Tchakarova SR, et al. Development of anti-Crimean-Congo hemorrhagic fever virus Gc and NP-specific ELISA for detection of antibodies in domestic animal sera. Front Vet Sci. 2022;9:913046. PubMed https://doi.org/10.3389/fvets.2022.913046

11. El Ghassem A, Apolloni A, Vial L, Bouvier R, Bernard C, Khayar MS, et al. Risk factors associated with Crimean-Congo hemorrhagic fever virus circulation among human, livestock and ticks in Mauritania through a one health retrospective study. BMC Infect Dis. 2023;23:764. PubMed https://doi.org/10.1186/s12879-023-08779-8

12. Negredo A, Sánchez-Arroyo R, Díez-Fuertes F, De Ory F, Budiño MA, Vázquez A, et al. Fatal case of Crimean-Congo hemorrhagic fever caused by reassortant virus, Spain, 2018. Emerg Infect Dis. 2021;27:1211–5. PubMed https://doi.org/10.3201/eid2704.203462

13. Lumley T. Analysis of complex survey samples. J Stat Softw. 2004;9:1–19. https://doi.org/10.18637/jss.v009.i08

14. QGIS.org. QGIS version 3.16.0 [cited 2022 May 1]. https://qgis.org

15. R Core Team. R: A language and environment for statistical computing. Vienna: The R Foundation for Statistical Computing; 2021.

16. Bates D, Mächler M, Bolker B, Walker S. Fitting linear mixed-effects models using {lme4}. J Stat Softw. 2015;67:1–48. https://doi.org/10.18637/jss.v067.i01

17. Bivand RS, Wong DWS. Comparing implementations of global and local indicators of spatial association. Test. 2018;27:716–48. https://doi.org/10.1007/s11749-018-0599-x

Appendix Table 1. Manufacturer-reported sensitivity and specificity values for the antigen test used in this study*

Species	% Specificity (95% CI)	% Sensitivity (95% CI)
Cattle	100 (99.1–100), n = 402	97.9 (92.6–99.4), n = 95
Sheep	100 (99.1–100), n = 402	99.0 (94.7–99.8), n = 102
Goats	100 (99.1–100), n = 402	100 (95.1–100), n = 74
Humans	100 (98.5–100), n = 257	NA

*ID Screen CCHF Double Antigen Multi-species (IDvet, Grabels, France). NA, not available.

Appendix Table 2. Odds of exposure to Crimean-Congo hemorrhagic fever in sheep and goats compared to cattle*

Variable	Odds ratio (95% CI)	p value
Intercept	0.75 (0.41–1.36)	0.341
Species		
Cattle	Referent	Referent
Goat	0.45 (0.39–0.51)	<0.001
Sheep	0.32 (0.27–0.37)	<0.001

*Odds ratio, 95% CIs, and p values from an all-species mixed effect logistic regression model with species as a fixed effect, and village and household as random effects.

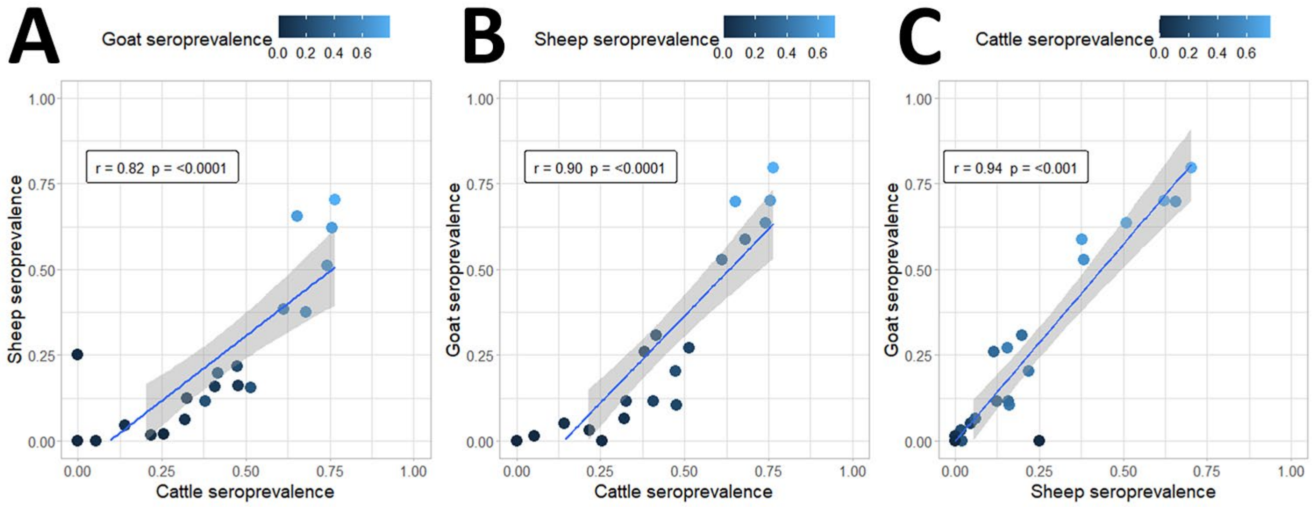

Appendix Figure 1. Correlation between village-level seroprevalence by species-pairs. A) Cattle and sheep seroprevalence compared with goat seroprevalence; B) cattle and goat seroprevalence compared with sheep seroprevalence; C) sheep and goat seroprevalence compared with cattle seroprevalence. Points are colored according to the village-level log odds of the third species.

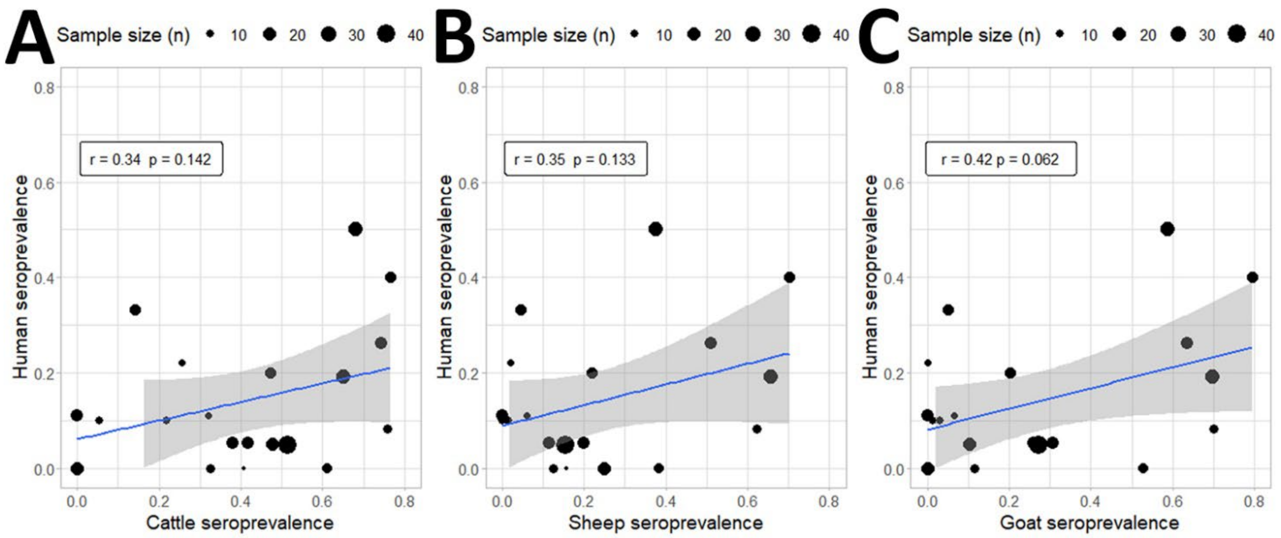

Appendix Figure 2. Correlation between village-level human and livestock seroprevalence. Graphs show human seroprevalence compared with seroprevalence of cattle (A), sheep (B), and goat (C) seroprevalence. Points are sized according to the village-level sample size.
